# Venomic, Transcriptomic, and Bioactivity Analyses of *Pamphobeteus verdolaga* Venom Reveal Complex Disulfide-Rich Peptides That Modulate Calcium Channels

**DOI:** 10.3390/toxins11090496

**Published:** 2019-08-27

**Authors:** Sebastian Estrada-Gomez, Fernanda Caldas Cardoso, Leidy Johana Vargas-Muñoz, Juan Carlos Quintana-Castillo, Claudia Marcela Arenas Gómez, Sandy Steffany Pineda, Monica Maria Saldarriaga-Cordoba

**Affiliations:** 1 Programa de Ofidismo/Escorpionismo—Serpentario, Universidad de Antioquia UdeA, Carrera 53 No 61–30, Medellín, Antioquia, CO 050010, Colombia; 2 Facultad de Ciencias Farmacéuticas y Alimentarias, Universidad de Antioquia UdeA, Calle 70 No 52–21, Medellín, Antioquia, CO 050010, Colombia; 3 Institute for Molecular Bioscience, The University of Queensland, 306 Carmody Road, St Lucia, QLD 4072, Australia; 4 Facultad de Medicina, Universidad Cooperativa de Colombia, Calle 50 A No 41–20 Medellín, Antioquia, CO 050012, Colombia; 5 Grupo de Génetica, Regeneración y Cáncer, Universidad de Antioquia UdeA, Carrera 53 No 61–30, Medellín, Antioquia, CO 050010, Colombia; 6 Brain and Mind Centre, University of Sydney, Camperdown, NSW 2052, Australia; 7 Garvan Institute of Medical Research, Darlinghurst, Sydney, NSW 2010, Australia; 8 St. Vincent’s Clinical School, University of New South Wales, Sydney, NSW 2010, Australia; 9 Centro de Investigación en Recursos Naturales y Sustentabilidad, Universidad Bernardo O’Higgins, Avenida Viel 1497, Santiago 7750000, Chile

**Keywords:** theraphosidae, *Pamphobeteus*, peptides, disulfide-rich peptide (DRP), inhibitory cysteine knot (ICK), venomics, transcriptome, ion channels

## Abstract

*Pamphobeteus verdolaga* is a recently described Theraphosidae spider from the Andean region of Colombia. Previous reports partially characterized its venom profile. In this study, we conducted a detailed analysis that includes reversed-phase high-performance liquid chromatography (rp-HPLC), calcium influx assays, tandem mass spectrometry analysis (tMS/MS), and venom-gland transcriptome. rp-HPLC fractions of *P. verdolaga* venom showed activity on Ca_V_2.2, Ca_V_3.2, and Na_V_1.7 ion channels. Active fractions contained several peptides with molecular masses ranging from 3399.4 to 3839.6 Da. The tMS/MS analysis of active fraction displaying the strongest activity to inhibit calcium channels showed sequence fragments similar to one of the translated transcripts detected in the venom-gland transcriptome. The putative peptide of this translated transcript corresponded to a toxin, here named ω-theraphositoxin-Pv3a, a potential ion channel modulator toxin that is, in addition, very similar to other theraphositoxins affecting calcium channels (i.e., ω-theraphotoxin-Asp1a). Additionally, using this holistic approach, we found that *P. verdolaga* venom is an important source of disulfide-rich proteins expressing at least eight superfamilies.

## 1. Introduction

Spider venoms are a complex mixture of neurotoxins, enzymes, proteins, antimicrobial, neurotoxic and cytolytic peptides, nucleotides, salts, amino acids, and neurotransmitters [1,2,3,4,5,6]. The production of this arsenal “manufactured” in the venom glands can be divided into three major groups: low (<1 kDa), medium (<10 kDa), and high (>10 kDa) molecular weight compounds. Medium molecular weight compounds correspond mainly to peptides that enhance neurotoxic activity. Their molecular masses range from 3 to 10 kDa, and their main targets are voltage-dependent sodium (Na_V_), potassium (K_V_), and calcium (Ca_V_) channels as well as calcium and potassium ligand-dependent channels (pre- and post-synaptic) and cholinergic receptors [1,2,3,4,5,6,7]. Although spider venom seems to be an important source of toxins modulating Ca_V_ channels [8], there are no reports describing these types of toxins in *P. verdolaga* venom.

Disulfide-rich peptides (DRPs) adopting the inhibitory cysteine knot fold comprise the most abundant components in spider venoms and are responsible for their toxic activities [9,10]. This inhibitory cysteine knot (ICK) scaffold has a key role in the structure/function and neurotoxicity, and it is defined as antiparallel β sheets stabilized by a cysteine knot formed by three disulfide bridges [9]. Additionally, the ICK motif can confer structural rigidity to maintain the active conformation for receptor binding and improved stability against thermal and enzymatic degradation [3].

Most ICK peptides described to date have been isolated mainly from the mygalomorphae suborder [9], specially from the Theraphosidae family, and in several genera including *Acanthoscuria*, *Chilobrachys*, *Grammosotola*, *Cyriopagopus* (*Haplopelma*), *Davus,* and *Thrixopelma*, with the majority of them having different pharmacological activities and molecular masses ranging from 3 to 5 kDa [11,12,13,14,15,16]. The main targets of these peptides include calcium-activated potassium (KCa) channels, voltage-gated calcium (Ca_V_) channels, voltage-gated sodium (Na_V_) channels and voltage-gated potassium (K_V_) channels [3,4,8,15,16].

A previous report from *P. verdolaga* showed that this venom contains low and high molecular mass proteins (i.e., phospholipase A_2_, sphingomyelinase D, hyaluronidase, Kunitz-type serine protease inhibitors, and compounds similar to lycotoxins) [17]. Several ICK fragments that matched theraphotoxins and affected voltage-gated calcium channels (U2-theraphotoxin-Asp1a and ω-theraphotoxin-Hh1a) were also described in this venom [18]. However, their full-length sequences and bioactivities remain unknown. In this study, we further characterized *P. verdolaga* venom and showed that, by using a combined approach, we were able describe a number of disulfide-rich peptide superfamilies with strong activity to inhibit Ca_V_ channels.

## 2. Results

### 2.1. rp-HPLC Profile

**Figure 1:**
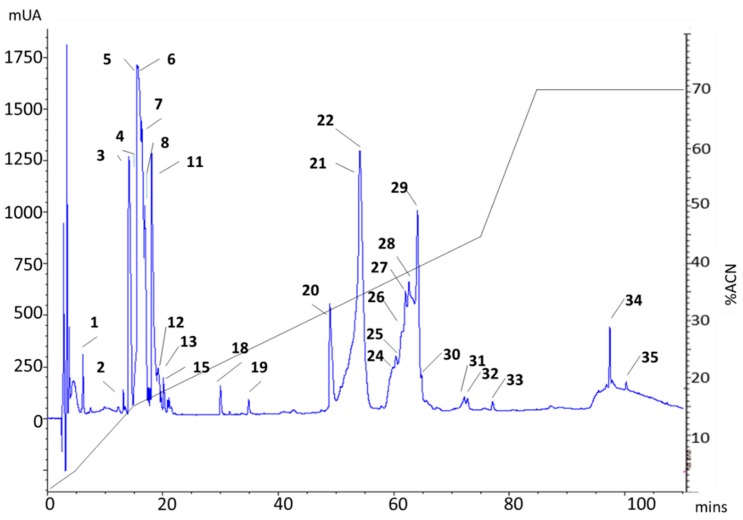
*Pamphobeteus verdolaga* venom reversed-phase high-performance liquid chromatography (rp-HPLC) chromatographic profile of 1 mg of crude venom using a C18 column (250 × 4.6 mm). The run was monitored at 215 nm using a flow rate of 1 mL/min and a gradient of 5% B for 5 min, followed by a linear gradient of 5%–15% B for 10 min, 15%–45% B for 60 min, and 45%–70% B for 12 min. Fractions collected are numbered/labeled on the top of the peaks.

rp-HPLC fractionation of crude *P. verdolaga* venom yielded a total of 35 fractions divided into two main regions, eluting between 15%–20% and 35%–40% of acetonitrile (ACN), as shown in Figure 1. To test the bioactivity of *P. verdolaga* venom, fractions from five different rp-HPLC runs were collected and analyzed using calcium and sodium channel assays. All runs showed the same chromatographic profile as observed in Figure 1. In some cases, fractions with large elution volumes were separated into different RNA-free plastic vials. To perform the mass spectrometry (MS/MS) analysis of active fractions, an additional run was carried out to collect the fraction of interest.

### 2.2. Bioactivity of Pamphobeteus verdolaga Venom

**Figure 2:**
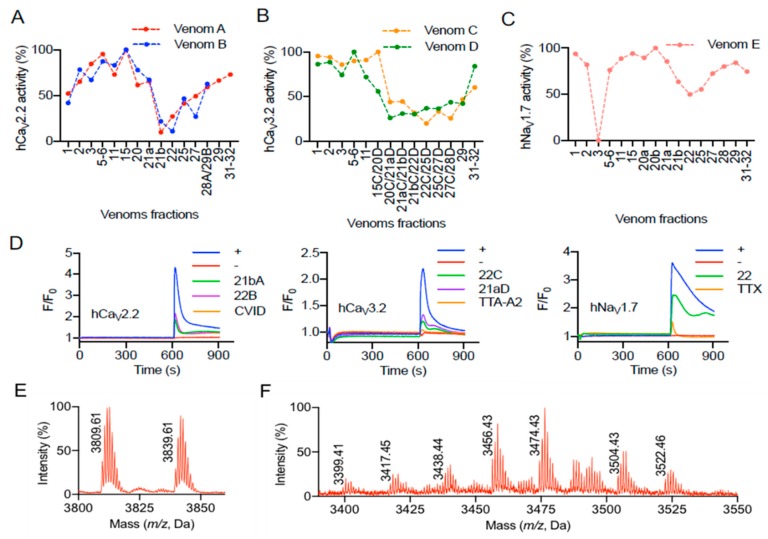
Bioactivity of the venom of *Pamphobeteus verdolaga*. Venoms fractions were tested for modulation of voltage-gated calcium and sodium channels. (**A**) Venoms A and B were evaluated for bioactivity on hCa_V_2.2, revealing that the fractions 21b and 22 had the strongest inhibitory effect. (**B**) Venoms B and C were evaluated for bioactivity on hCa_V_3.2, revealing that the fractions 21a and 22 had the strongest inhibitory effect. (**C**) Venom E was evaluated for bioactivity on hNa_V_1.7, revealing the fractions 3 and 22 had the strongest inhibitory effects. (**D**) Fluorescent traces of the calcium influx assay showed responses over baseline for the entire time course of the bio-assay. Positive and negative controls are represented in blue and red lines, respectively, and specific inhibition controls are in orange. Calcium response traces are represented in green for venom A fraction 21b (21bA) and purple for venom B fraction 22 (22B) in the hCa_V_2.2 assay, green for venom C fraction 22 (22C) and purple for venom D fraction 21a (21aD) in the hCa_V_3.2 assay, and in green for venom E fraction 22 in the hNa_V_1.7 assay. (**E**,**F**) Mass spectrometry analysis using matrix-assisted laser desorption/ionization time-of-flight (MALDI-TOF) of the fractions 21a-b (**E**) and 22 (**F**) with strong inhibition of hCa_V_2.2 and hCa_V_3.2, and approximately 50% inhibition of hNa_V_1.7, respectively.

Spider venoms are known for their exquisite ability to modulate voltage-gated ion channels [10]. The bioactivities of *P. verdolaga* venom fractions were evaluated in endogenously expressed voltage-gated sodium and calcium channels in neuroblastoma cell lines or recombinantly expressed in HEK293T cells. Calcium influx assays revealed that the venom was able to inhibit hCa_V_2.2, hCa_V_3.2, and hNa_V_1.7 responses (Figure 2). Venom fractions (according to Figure 1) from five independent fractionations by rp-HPLC (venom fractionations A to E) were assayed for bioactivity. Venom fractionations A and B were evaluated for their activity on hCa_V_2.2 (in duplicate), showing that fractions 21b and 22, corresponding to elution times of 54 and 55 min and 33% and 34% B, respectively, had the strongest inhibitory activities (Figure 2A). Venom fractionations C and D were evaluated for their activity on hCa_V_3.2 (in duplicate), showing that fractions 21a and 22, corresponding to elution times of 53 and 55 min and 32% and 34% B, respectively, had the strongest inhibitory activities (Figure 2B). Venom fractionation E was evaluated for its activity on hNa_V_1.7, showing that fractions 3 and 22, corresponding to elution times of 14 and 55 min and 14% to 34% B, respectively, had the strongest inhibitory activities (Figure 2C). Fluorescent traces from the calcium influx assays showed no increase in intracellular Ca^2+^ upon the addition of the venom fractions, which suggests the observed activities were only inhibitory (Figure 2D).

Mass spectrometry (MS) analyses of fractions 21a-b, 22, and 25, using matrix-assisted laser desorption/ionization time-of-flight (MALDI-TOF), showed strong inhibition of hCa_V_2.2 and hCa_V_3.2 and approximately 50% inhibition of hNa_V_1.7. The MALDI trace also showed multiple masses ranging from 3399.4 to 3839.61 Da (Figure 2E,F). The peptide masses obtained from the MS analysis revealed a similar mass of 3810.6 Da in fraction number 25 (data not shown), which was approximately 1 Da different from the mass 3809.61 Da found in the Ca_V_ bioactive fractions. This suggests the native peptide displaying 3809.61 Da is the C-terminal amide form of the 3810.6 Da peptide. C-terminal amidation is a common post-translational modification found in spider ICK peptides and usually confers stronger bioactivity compared to the respective C-terminal carboxy version [15,19]. Further, venom E fraction 3 did not show any masses above 1000 Da, which suggests the inhibition of Na^2+^ influx responses observed was induced by polyamines [20] or other small molecules present in larger quantities in venom E compared to venoms A to D. Also in venom E, the masses 3809.61 and 3839.61 Da were found at high intensities in fractions 21a and 21b, and with less intensity in fractions 25 and 27, suggesting these newly found peptides had a weak effect in the hNa_V_1.7 activity tested in our bio-assays.

### 2.3. Tandem Mass Spectrometry (MS)/MS Analysis of the Active Fraction

**Figure 3:**
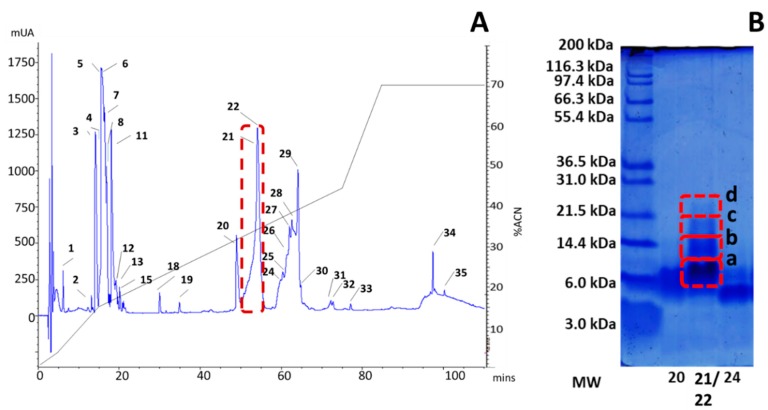
(**A**) The single peak from *P. verdolaga* venom highlighted in red boxes, along with sodium and calcium active fractions. (**B**) Active fractions Tris-Tricine gels showing the overall sizes of different peptides that were subjected to automated reduction/alkylation with DTT and iodoacetamide. Peptides were digested with porcine trypsin using a ProGest™ digestor.

Active fractions 21 and 22 (according to Figure 1) were collected in a single RNA-free plastic vial and subjected to tandem mass spectrometry (tMS/MS). The separation of venom components using Tris-Tricine gels showed that the likely active fractions of *P. verdolaga* venom corresponded to components with a molecular mass ranging from 6 to 21 kDa (Figure 3B, each red box highlights bands a, b, c, and d). Mass spectrometric analysis of the in-gel digestion of protein bands revealed the presence of 3 fragments that matched peptides with molecular masses below 6 kDa. No fragments with molecular masses similar to high molecular mass compounds (HMMCs), above 10 kDa, were availably detected, although the Tris-Tricine gel allowed the detection of different bands corresponding to these HMMCs.

tMS/MS analysis of in-gel digestion of protein bands separated by Tris-Tricine allowed the detection of four different fragment sequences (see Table 1). After a local protein Basic Local Alignment Search Tool (BLASTP) analysis using the translated transcriptomic information of *P. verdolaga* venom gland as the local database, tMS/MS sequences showed hits exhibiting, in some cases, a resemblance identity of 100% with translated transcripts (see Table 1). The transcriptomic analysis allowed the detection of a sequence that matched 66% of the MS/MS results (see below).

### 2.4. Transcriptomic Results

#### 2.4.1. General Transcriptomic Assembly Analysis

A de novo reference transcriptome of *P. verdolaga* was generated from the RNAs isolated from the venom gland. A total 46,598,494 high-quality pair-end reads were assembled using Trinity v.2. A total of 78,088 contigs were obtained with a GC content of 39.19%, with an average and maximum contig length of 596 and 13,249 base pairs (bp), respectively. The length distribution ranged from 201 to 13,249 bp, 68% < 500 bp, 18% 500–1000 bp, and 14% > 1000 bp (Supplementary File S1). Based on read coverage, the E90N50 statics showed ~1300 bp (Supplementary File S1), and the reference transcriptome contained 79.8% of the highly conserved sequences among arthropods (850 out of 1066) and 84.2% (255 out of 303) among eukaryotes.

#### 2.4.2. Transcriptomic Annotation

**Figure 4:**
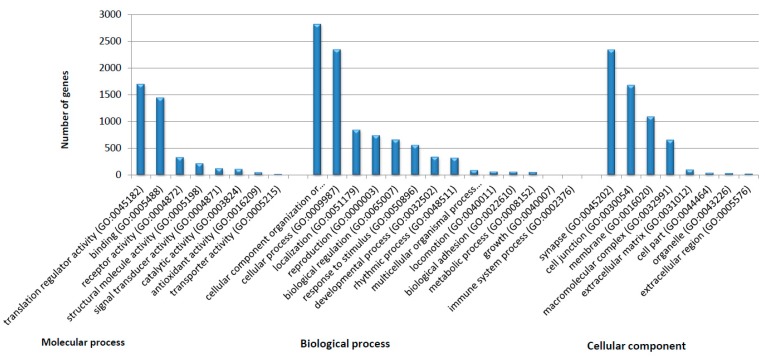
Gene ontology (GO) categories for annotated *Pamphobeteus verdolaga* transcripts. The graph shows the Level 2 categories detected for biological processes, molecular function, and cellular components.

The reference transcriptome of the venom gland from *P. verdolaga* was annotated with the Trinotate pipeline (https://github.com/Trinotate/Trinotate). Utilizing this pipeline, a total of 16,030 transcripts were annotated (Supplementary file 2), and, subsequently, the list of nonredundant genes (*n* = 7137 genes) was clustered in gene ontology (GO) categories (Figure 4). The main biological process was cellular component organization, or biogenesis (GO:0071840), the most representative molecular process was translation regulator activity (GO:0045182), and the principal cellular component was synapse (GO:0045202).

**Figure 5:**
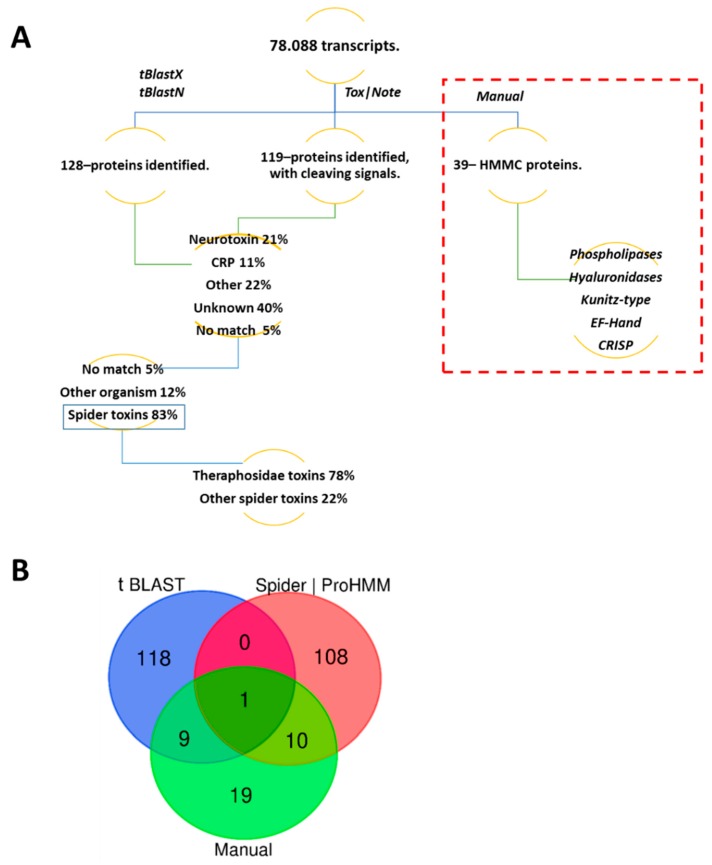
Strategies used for toxin identification based on transcriptomic data. (**A**) Figure shows the relative proportion of the transcripts identified in the venom gland of *P. verdolaga* following a BLAST search using tBLASTN, tBLASTX, and Tox|Note. Dashed boxes show the relative proportion of the transcripts identified by manual analysis. (**B**) Venn diagram showing the degree of overlap between all strategies used.

After acquiring GO annotation for all transcripts, we used a three-step approach to extract the maximum number of sequences containing hits to toxin/toxin-like transcripts and venom protein transcripts. Briefly, we used, as the first step, a search that utilized Basic Local Alignment Search Tool searching translated nucleotide databases using a translated nucleotide and protein query (tBLASTX and tBLASTN). Using this strategy, a total of 128 sequences were identified. Similarly, using the Tox|Note pipeline, a total of 119 peptides were identified. Furthermore, Tox|Note provided important information about the processing predictions for peptides. Finally (and additionally), using a manual directed search, a total of 39 sequences were identified. The majority of these sequences corresponded mainly to high molecular mass compounds (HMMCs) and included: phospholipases, hyaluronidases, Kunitz-type peptides, EF-hand proteins. and cysteine-rich secretory proteins (CRISP; data not shown). The Venn diagram shows the coincidence detected in each analysis with a final count of 265 different hits (see Figure 5).

From the 265 contigs identified using tBLASTX, tBLASTN, and SpiderPro|HMM, 21% corresponded to hits with known neurotoxins affecting different ion channels (NTAIC); 11% corresponded to cysteine-rich proteins (CRPs) like theraphotoxins, hainantoxins, huwetoxins (different to CRISP), or colipase-like proteins; 22% showed different biological activities (i.e., hyaluronidases, calcium-binding (EF-hand), and serine protease inhibitor); 40% were toxins with unknown activity; and 5% did not show a match (Figure 3). Eighty-three percent of these contigs are similar to spider proteins, and 78% of transcripts showed similitude with spider proteins corresponding to proteins reported in the Theraphosidae family (Figure 5).

**Figure 6:**
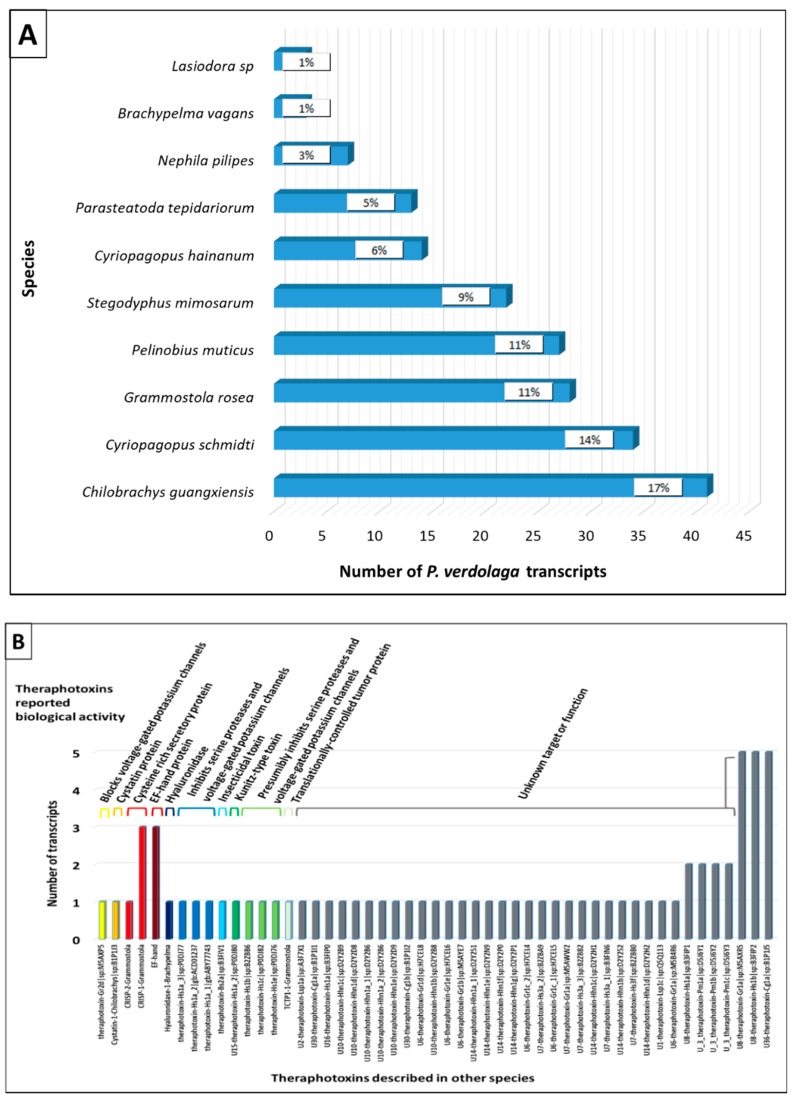
Similarity of *P. verdolaga* transcripts identified using tBLAST or Tox|Note with: (**A**) other spiders venom contents and (**B**) theraphotoxins.

The translated and identified transcripts also showed high similarity with toxins that have been previously reported in the Theraphosidae family. Most of the transcripts displayed similarity with toxins isolated from *Chilobrachys guangxiensis*, *Cyriopagopus schmidti* (*H. schmidti*), *Cyriopagopus hainanus* (*H. hainanum*), *G. rosea,* and *Pelinobius muticus* (Figure 6A). Seventy-one translated transcripts showed homology with different theraphotoxins known to have insecticidal activity and block/modulate potassium channels. Seventy-three percent of the theraphotoxins had an unknown target or function (Figure 6B).

Twenty-six translated transcripts were classified as CRP molecules and were divided into 8 superfamilies according to their cysteine pattern, as summarized in Table 2. Length and number of cysteines differ between Superfamilies, and only two showed the common inhibitory cystine knot (ICK) motif. A detailed description of each superfamily is described in the following section.

##### Superfamily 1

This group included only one sequence characterized by the presence of 5 cysteine residues and a length of 54 amino acids. The predicted disulfide pattern suggested that cysteine bond connectivities were between CysII–CysIII and CysIV–CysV, with cysteine I not forming any bridge. This protein is similar to the U35-theraphotoxin-Cg1a (B1P1J4) from *C. guangxiensis* with an *e*-value of 7 × 10^−24^. The activity of this peptide is still unknown.

##### Superfamily 2

Group 2 included one sequence with an amino acid length of 48 residues and 6 cysteines. The disulfide bond pattern was predicted between amino acids CysI–CysIV, CysII–CysV, and CysIII–CysVI. This peptide is similar to a hypothetical protein reported on the Theraphosidae spider *Parasteatoda tepidariorum* with an *e*-value of 1 × 10^−13^ and with an unknown biological activity.

##### Superfamily 3

Two sequences were included in this group characterized by 7 cysteine residues and three disulfide bridges formed between CysII–CysV, CysIII–CysVI, and CysIV-CysVII, with cysteine I not forming a bridge. SF3 members had between 43 and 84 residues. Both peptides from this group did not show similarity with any known protein.

##### Superfamily 4

**Figure 7:**
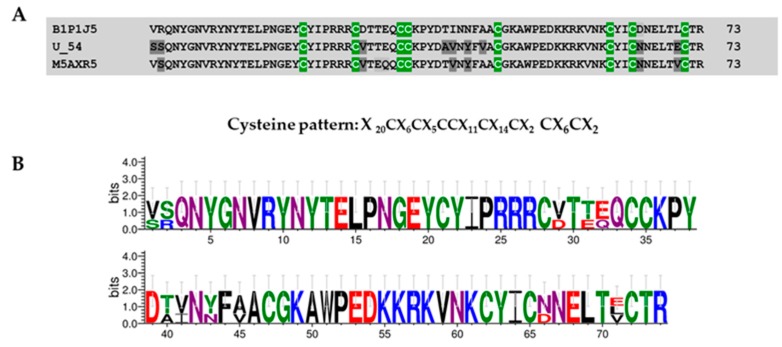
Multiple alignment of cystine knot toxins (CKTs) present in *Pamphobeteus verdolaga* venom with CKT previously characterized (**A**) and sequence logos (**B**). Group 4a U54-theraphositoxin-Pv1a_1 putative toxin precursor present similitude with *Grammostola rosea* GTx6-1, M5AXR5, and Cystine knot toxins of the tarantula *Chilobrachys jinzhao* assigned by Chen et al. 2008 in orphan family 2 (JZTX-72; B1P1J5). Polymorphic sites are indicated in gray.

Four sequences were identified in this group displaying a length between 44 and 74 residues. This family was characterized by the presence of 4 disulfide bridges between either cysteines CysI–CysVIII, CysII–CysIII, CysIV–CysV, and CysVI-CysVII or CysI–CysVII, CysII–CysVIII, CysIII–CysVI, and CysIV–CysV. Members of this knottin group, for example, U54-theraphositoxin-Pv1a_1, a putative toxin precursor that has a similar cysteine pattern (“X_20_CX_6_CX_5_CCX_11_CX_14_CX_2_CX_6_CX_2_”; X is any amino acid), showed similarity with the cystine knot toxin and domains of *G. rosea* GTx6-1 (M5AXR5) and to cysteine knot toxins of the tarantula *C. jinzhao* (“*guangxiensis*”), assigned by Chen et al. (2008) in orphan family 2 (JZTX-72; B1P1J5) (see Figure 7) [12]. This cluster of toxins was named group 4a.

**Figure 8:**
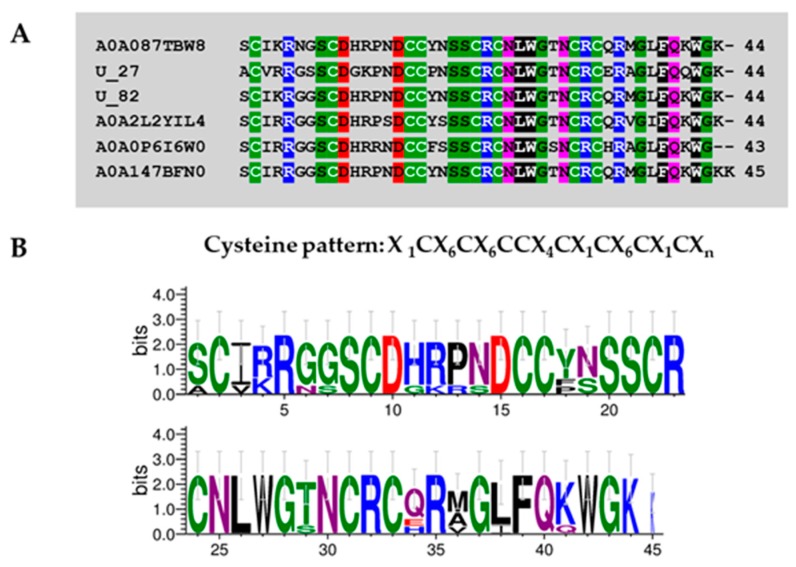
Multiple alignment of spider toxins present in *Pamphobeteus verdolaga* venom with agatoxin previously characterized (**A**) and sequence logos (**B**). Group 4b U27-theraphositoxin-Pv1a_1 and U82-theraphositoxin-Pv1a_1 putative toxin precursors present similitude with U8-agatoxin-Ao1a from *Stegodyphus mimosarum* (A0A087TBW8), *Parasteatoda tepidariorum* (A0A2L2YIL4, house spider), *Daphnia magna* (A0A0P6I6W0), and putative U8-agatoxin-ao1a-like isoform x2 from *Ixodes ricinus* (A0A147BFN0, common tick).

In addition, putative toxin precursors U27-theraphositoxin-Pv1a_1 and U82-theraphositoxin-Pv1a_1 had similar cysteine patterns (“X_1_CX_6_CX_6_CCX_4_CX_1_CX_6_CX_1_CX_n_”; X is any amino acid, and n is an uncertain number) to the spider toxin U8-agatoxin-Ao1a from *Stegodyphus mimosarum* (A0A087TBW8) and *Parasteatoda tepidariorum* (A0A2L2YIL4, house spider). Furthermore, they showed similarity with the U8-agatoxin-Ao1a from *Daphnia magna* (A0A0P6I6W0) and putative U8-agatoxin-ao1a-like isoform x2 from *Ixodes ricinus* (A0A147BFN0, common tick) (see Figure 8). This cluster of toxins was named group 4b.

##### Superfamily 5

This family included two sequences characterized by the presence of 8 cysteine residues and an amino acid length between 61 and 65 residues. The predicted disulfide pattern suggested that cysteine bonds formed between CysI–CysVI, CysII–CysVII, CysIII–CysIV, and CysV-CysVIII. Both peptides from this family did not show any similarity with any known peptides.

##### Superfamily 6

**Figure 9:**
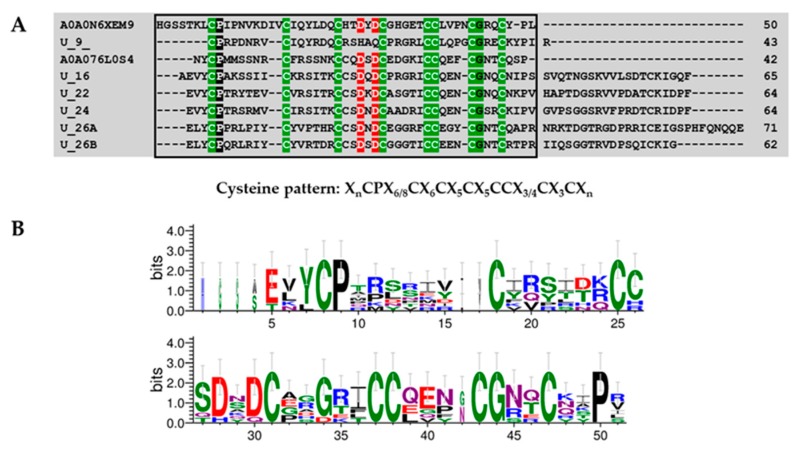
Multiple alignment of whey acid protein (WAP)-type toxins present in *Pamphobeteus verdolaga* venom with WAP protein previously characterized (**A**) and sequence logos (**B**). Group 6 U9, U16-theraphositoxin-Pv1a_1, U22-theraphositoxin-Pv1a_1, U24-theraphositoxin-Pv1a_1, U26-theraphositoxin-Pv1a_1, and U26-theraphositoxin-Pv1b_1 putative toxin precursors are similar to *Eriocheir sinensis* (A0A0N6XEM9) and *Nephila pilipes* (A0A076L0S4). The WAP-type ‘four-disulfide core’ domain profile is indicated in the box.

Six peptides were identified in this group, with amino acid lengths between 43 and 71 residues, and were predicted to form four disulfide bridges. Cysteine bond formations were predicted between CysI–CysVI, CysII–CysVII, CysIII–CysV, and CysIV–CysVIII. All members of this group displayed the whey acid protein (WAP)-type four-disulfide core domain with a cysteine pattern (“X_n_CPX_6/8_CX_6_CX_5_CX_5_CCX_3/4_CX_3_CX_n_”; X is any amino acid and n is an uncertain number) similar to WAP domain identify in *Eriocheir sinensis* (A0A0N6XEM9) and *Nephila pilipes* (A0A076L0S4) (Figure 9).

##### Superfamily 7

**Figure 10:**
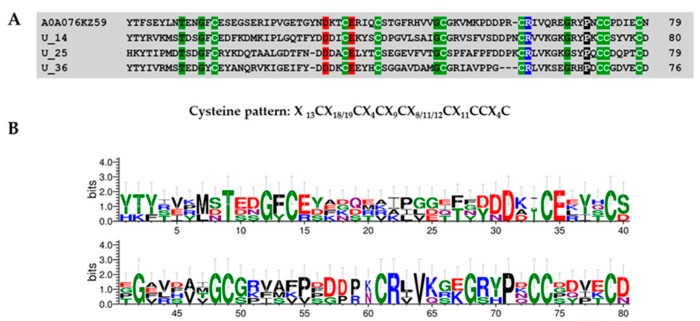
Multiple alignment of single-domain von Willebrand factor type C (SVWC) domain toxins present in *Pamphobeteus verdolaga* venom with SVWC protein previously characterized (**A**) and sequence logos (**B**). Group 7 U14-theraphositoxin-Pv1a_1, U25-theraphositoxin-Pv1a_1, and U36-theraphositoxin-Pv1a_1 putative toxin precursors are similar to *Nephila pilipes* (A0A076KZ59).

This group was characterized by 2 proteins with amino acid lengths between 76 and 80 residues and the presence of 4 disulfide bridges between cysteines CysI–CysV, CysII–CysVI, CysIII–CysVII, and CysIV-CysVIII. All members of this group displayed the single domain von Willebrand factor type C domain with a cysteine pattern (“X_13_CX_18/19_CX_4_CX_9_CX_8/11/12_CX_11_CCX_4_C”; X is any amino acid) similar to SVWC domain identify in *Nephila pilipes* (A0A076KZ59) (Figure 10).

##### Superfamily 8

**Figure 11:**
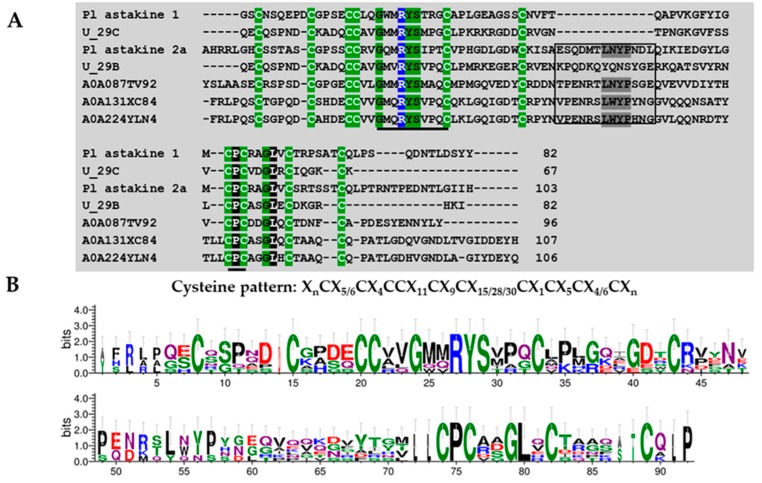
Multiple alignment of astakine isoforms present in *Pamphobeteus verdolaga* venom with astakine previously characterized (**A**) and sequence logos (**B**). Group 8 U29-theraphositoxin-Pv1c_1 is similar to the previously isolated Pl astakine 1 from *Pacifastacus leniusculus* (Q56R11). U29B-theraphositoxin-Pv1b_1 is similar to the previously isolated compound. Pl astakine 2a from Pacifastacus leniusculus (A5HTU2), Stegodyphus mimosarum (A0A087TV92), Hyalomma excavatum (A0A131XC84) and Rhipicephalus zambeziensis (A0A224YLN4, Ixodegrin B). U_29C-theraphositoxin-Pv1c_1 like Pl astakine 1 have 15 amino acid residues between C#6 and C#7. U29B like Pl astakine 2a have an insert of 13 amino acid residues between C#6 and C#7 (Boxed). The conserved motifs GX2RYSX(P/R/A)XC and CPC are underlined. Conserved LXYP motif in astakine.

This group included eight sequences characterized by the formation of 5 disulfide bridges and amino acid lengths between 67 and 88 residues. Predicted disulfide bounds were either between CysI–CysVI, CysII–CysIV, CysIII–CysV, CysVII–CysX and CysVIII–CysIX or CysI–CysIII, CysII–CysIV, CysV–CysVI, CysVII–CysVIII, and CysIX–CysX. Six members from this group did not show similarity with any known domain. Only two putative toxin precursors had similar cysteine patterns (“XnCX5/6CX4CCX11CX9CX15/28/30CX1CX5CX4/6CXn”; X is any amino acid and n is an uncertain number) with other identified invertebrates and spider toxin cytokines as astakines (Figure 11). U29-theraphositoxin-Pv1c_1 is similar to the previously isolated Pl astakine 1 from *Pacifastacus leniusculus* (Q56R11), while U29-theraphositoxin-Pv1b_1 is similar to the previously isolated Pl astakine 2a isolated from *Pacifastacus leniusculus* (A5HTU2), *Stegodyphus mimosarum* (A0A087TV92), *Hyalomma excavatum* (A0A131XC84), and *Rhipicephalus zambeziensis* (A0A224YLN4, Ixodegrin B). The U29C-like Pl astakine 1 has 15 amino acid residues between C#7 and C#8. The U29-theraphositoxin-Pv1b_1-like Pl astakine 2a has an insert of 13 amino acid residues between C#7 and C#8 (Boxed in Figure 11). The motif GX2RYSX(P/R/A) XC was found in both toxin precursors isolated in this study and all invertebrate sequences shown in Figure 10. The LXYP motif present in 2 was not present in U29-theraphositoxin-Pv1b_1.

#### 2.4.3. Transcriptomics Reveal Sequences of Potential Voltage-Gated Calcium Channel Modulators

**Figure 12:**
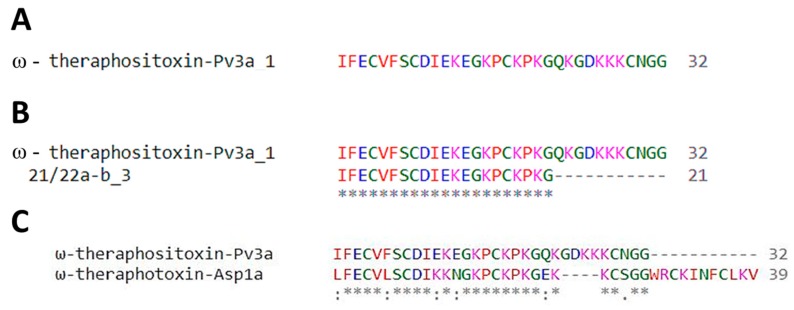
(**A**) ω-theraphositoxin-Pv3a mature sequence derived from the combination of the tandem MS/MS analysis and venom-gland transcriptomics. (**B**) Pairwise alignment of ω-theraphositoxin-Pv3a and the tandem MS/MS fragments. Asterisks (*) indicate an identical match. (**C**) Pairwise alignment of ω-theraphositoxin-Pv3a and ω-theraphositoxin-Pv3a-Asp1a from *Aphonopelma* sp. (proved theraphotoxin to inhibit voltage gated calcium channels)**.**

After a local BLAST using the venom-gland transcriptomic results as the database and the tMS/MS fragments as the query, all hits were similar to transcript c7142 (c7142_g1_i1). This transcript, with a transcripts per million (TPM) value of 27806.19, corresponds to a sequence that is 82 residues long with a theoretical molecular weight of 8896.18 Da. After processing the signal and propetide cleaving sites, the mature peptide of c7142 is a 32 residue peptide with a theoretical monoisotopic molecular weight of 3495.69 Da (considering all cysteines are oxidized) and is named ω-theraphositoxin-Pv3a (Figure 12A). MALDI-TOF MS analyses of voltage-gated active fractions showed a molecular mass range between 3399.41 and 3839.61 Da (Figure 2E, F). In an attempt to determine the complete sequence of ω-theraphositoxin-Pv3a, we combined the tMS/MS analysis and venom-gland transcriptomics. Fragments corresponding to 21-22/a-b_1 matched the propeptide region (data not shown), while 21-22/a-b_2, 21-22/a-b_3 and 21-22/c_1 matched the ω-theraphositoxin-Pv3a_1, covering 66% of the toxin. Fragments 21-22/a-b_2 and 21-22/c_1 were contained in fragments 21-22/a-b_3 and were 100% identical (see Table 1). Figure 12B shows the pairwise sequence alignment of ω-theraphositoxin-Pv3a_1 sequence with the tMS/MS results of fraction 21/22. tMS/MS fractions did not show any similarities with any other transcribed transcript. This theraphositoxin from *P. verdolaga* was similar (e-value > 2 × 10^−4^) to other theraphositoxins reported in the Theraphosidae family, in the species (*Aphonopela* sp. and *californicum*), *Brachypelma* (*Brachypelma albicans* and *Brachypelma smithi*), and *Acanthoscuria paulensis*, all distributed in the American continent (United States, Mexico, and Brazil, respectively, according to [21]). ω-theraphositoxin-Pv3a shows high sequence similarity to ω-theraphotoxin-Asp1a (*e*-value of 2 × 10^−4^) from the related spider *Aphonopelma sp,* which is known and proved to inhibit voltage-gated calcium channels (see Figure 12C) [22,23,24]. It was similar as well (62.5% identity) with the calcium active peptide, identified in the theraphosid *Coremiocnemis tropix*. Nevertheless, the full sequence and bio-activity of Pv3a still has yet to be confirmed in further venomic, transcriptomic, and pharmacological studies.

**Figure 13:**
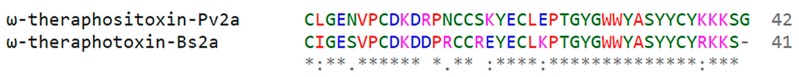
ω-theraphotoxin-Pv2a mature peptide sequence detected in the transcriptome analysis and pairwise alignment with ω-theraphotoxin-Bs2a from *Brachypelma smithi*.

Besides Pv3a, other theraphositoxins detected in the transcriptome showed high identity to calcium channel inhibitors such as ω-theraphotoxin-Bs2a (*e*-value: 3 × 10^−18^) isolated from *Brachypelma smithi* [25] (see Figure 13). This novel peptide has a theoretical monoisotopic mass of 4900 kDa (considering all cysteines are oxidized) and corresponds to a DRP named here ω-theraphositoxin-Pv2a.

## 3. Discussion

The complexity of Theraphosidae venoms is commonly underestimated because of the lack of high-throughput methods for toxin identification [14,26]. Venomics allows the study of this venom by integrating proteomic, genomic, and transcriptomic analyses [27]. To date, only a handful of Theraphosidae venoms have been extensively characterized, including the venoms of the Chinese black earth tiger tarantula *C. hainanus* (*H. hainanum*), *C. schmidti* (*H. schmidti*), and *C. guangxiensis*, among others [28]. *P. verdolaga* is a recently described Theraphosidae spider distributed mainly in the region of Antioquia (Colombia) [29]. MS/MS and transcriptomics analyses of the venom-gland content showed that *P. verdolaga* venom is not only composed of theraphotoxins, but also by larger proteins, in agreement with what has been previously reported for tarantulas [17].

Tarantula venoms are a rich source of bio-active molecules specialized in modulating ion channels [10,19]. Such peptides are believed to have an important role in spider behavior during prey capture and defense from predators. Indeed, the venom of *P. verdolaga* contained peptides that were able to modulate the ion channels hCa_V_2.2, hCa_V_3.2, and hNa_V_1.7. Interestingly, our bioactivity assay results suggest that the venom of *P. verdolaga* has the ability to preferentially modulate Ca_V_ channels over Na_V_ channels, although more detailed experiments are required to explore these observations further at the single peptide level using ion channel electrophysiology assays. These findings are in agreement with previous reports of *P. verdolaga* venom that described the presence of peptides with high identity with Ca_V_ channel modulators from other Theraphosids [17,18].

Disulfide-bonded neurotoxins are the most common toxins in spider venoms as well as in scorpion and cone snail venoms [1,3,11,12,13,14,28,30]. Interestingly, non-Theraphosidae spider peptides that potently inhibit Ca_V_2 channels often display disulfide bridge patterns other than a typical ICK scaffold, as reported for toxins in the venom of the genus *Phoneutria*, for example—ω-ctenitoxins Pn2a (Tx3-3) [31], Pn3a (Tx3-4) [32], Pn4A (Tx3-6) [33] as well as Pr1a (PRTx3-7) [34]—and for ω-segestritoxin of the genus *Segestria* [35]. Most of the groups of toxins found in the venom of *P. verdolaga* displayed disulfide patterns that resembled these Ca_V_2 inhibitory toxins. Bioactive results were consistent with the MS/MS findings, where the active fractions displaying the inhibitory activity on Ca_V_ channels possessed peptides showing homology with peptides assumed to inhibit voltage-gated calcium channels [22]. These findings encourage more detailed studies of the components of *P. verdolaga* venom to further characterize the peptides responsible for the interesting pharmacological effects observed. Only one fraction of *P. verdolaga* venom showed strong inhibitory activity on hNa_V_1.7. Interestingly, another species belonging to the genera *Pamphobeteus*, *P. nigricolor*, was previously described to contain bio-active peptides but with a strong and selective inhibition of Na_V_1.7 channels [18]. The tMS/MS analysis of the active fraction showed the presence of fragments similar to the mature peptide of transcribed transcript c7142. This mature peptide was named ω-theraphositoxin-Pv3a, which may be responsible for the activity on Cav channels. Additionally, the similarity of this theraphotoxin with ω-theraphotoxin-Asp1a, which is also known to affect voltage-gated calcium channel in vertebrates (based on sequence homologies), supports this hypothesis [8,22]. ω-theraphositoxin-Pv3a may correspond to a fragment of the mature peptide that is very similar to other theraphotoxins, like ω-theraphotoxin-Asp1a from *Aphonopelma sp* and Ct1a from *Coremiocnemis tropix* (both calcium active peptides), but missing at least two cysteines to allow the three disulfide bridges common in ICK peptides [36]. The full sequence and bio-activity of Pv3a is still to be confirmed in further venomic, transcriptomic, and pharmacological studies.

*P. verdolaga* transcriptomic results are in agreement with the Chen et al. report for the spider *C. hainanus* (*H. hainanum*) [14]. Both analyses showed the presence of CRISP, the Kunitz-type toxin, insecticidal toxins, and a significant number of proteins with unknown functions [14]. The last group of unknown proteins is of special interest since it could indicate several new variants of potential novel toxins as described in other Theraphosidae venoms [14]. Cystein-rich peptides forming cysteine knots, obtained from the transcriptome, are similarly clustered as described in the Theraphosidae spiders *G. rosea*, *C. schmidti* (*H. schmidti*), *C. guangxiensis*, and *C. hainanus* (*H. hainanum*) [12,14,28,37]. We found two different cytokines in *P. verdolaga* venom, astakine 1 and astakine 2, which have been found in several invertebrate species: astakine 1 in *Pacifastacus leniusculus* (crayfish), *Acanthoscurria gomesiana* (tarantula spider), and *Ornithoctonus huwena* (the Chinese bird spider) and astakine 2 in crustaceans, ticks, and spiders as well as in blattodean, hymenopteran, and hemipteran insects [38]. Surprisingly, both astakine sequences have been described as having different roles in hemocyte lineage differentiation and show conserved motifs among invertebrate groups. An interesting finding is that the astakine-2 transcript found in this study may not exhibit the same function in granular cell differentiation as previously described by Lin et al. 2010 [38], as the new sequence does not contain the conserved motif LXYP (L > Y; Y > N; P > S) required for that function. It is also known that when leucine (Leu72) and tyrosine (Tyr74) in this conserved motif are mutated into glycines in r-astakine 2Mut, its activity in promoting the maturation of granular cells is abolished, further offering support for the hypothesis that astrakine-2 has a different function.

## 4. Materials and Methods

### 4.1. Spider Collection and Venom Extraction

Five female *Pamphobeteus verdolaga* specimens were collected in the locality of La Estrella-Pueblo Viejo, Antioquia Province, Colombia. Venom from five specimens was obtained as previously described [18]. Venom extraction was carried out using electro-stimulation. Metal electrodes, impregnated with a saline solution, were carefully positioned on the chelicerae, and a block signal with an amplitude of 18 V at 40–60 Hz was applied twice with an interval of 5 s using a custom-made electro-stimulator (model 01). Collected venom was transferred to dry, low-protein binding vials, lyophilized, and stored at −20 °C until use. All tissue and venom collections were conducted in accordance with: (a) the ethical principles in animal research adopted by the World Health Organization for the characterization of venoms [39,40] and (b) the “Comité Institucional para el Cuidado y Uso de Animales” (CICUA). After each extraction, all animals were kept alive in captivity.

### 4.2. Venom Fractionation

The venom profile of *P. verdolaga* was obtained using reversed-phase high-pressure liquid chromatography (rp-HPLC). One milligram of crude venom was dissolved in 200 μL of solution A (0.1% TFA in water) and centrifuged at 3500× *g* for 5 min at room temperature. The supernatant was fractionated using a C_18_ rp-HPLC analytical column (250 × 4.6 mm), equilibrated and eluted at a flow rate of 1.0 mL/min, first isocratically (5% B for 5 min), followed by a linear gradient of 5%–15% B for 10 min, 15%–45% B for 60 min, and 45%–70% B for 12 min [41]. The chromatographic separation was monitored at 215 nm, and fractions were collected manually, lyophilized, and stored −20 °C until used. Chromatographic fractions were analyzed by gel electrophoresis using 10% Tris-Tricine gels or 12% sodium dodecyl sulfate polyacrylamide gels [42] as explained below. Genetic accession was carried out under contract 155 signed by the University of Antioquia with the Environmental Ministry of the Republic of Colombia.

### 4.3. Sample Preparation for Proteomic Analysis

Bands/spots selected for proteomic analysis were excised from Tris-Tricine gels and subjected to automated reduction/alkylation with DTT and iodoacetamide, respectively. Peptides were digested with porcine trypsin (Promega, Madison, WI, USA) using a ProGest™ digestor (Genomic solutions, model PRO100001, v2.00.07, Ann Arbor, MI, USA) following the manufacturer’s instructions. The dried digests were re-suspended in 8 μL of 0.1% formic acid and subjected to LC-MS/MS analysis using an aSynapt G2 ESI-QTOF instrument (Waters, Manchester, UK) (see below).

### 4.4. Data Analysis

MS/MS spectra were interpreted manually or using a licensed version of ProteinLynx Global (Server v2.5.2 software from Waters, Waters, Manchester, UK) or a free version of MASCOT (http://www.matrixscience.com). ProteinLynx searches were made using a tryptic digestion with two missed cleavages. The peptide tolerance was set to 10 ppm, while fragment tolerance and estimated calibration error were set to 0.05 and 0.005 Da, respectively. Carbamidomethyl cysteine and oxidation of methionine were fixed as well as variable modifications [43].

### 4.5. LC-MS/MS

Six microliter aliquots were subjected to nano-LC separation (nanoACQUITY-UPLC, Waters, Manchester, UK) equipped with a C18 (100 μm × 100 mm, 1.7 μm particle size) BEH130 column operated at 0.6 μL/min and coupled to ESI-Q-TOF (Synapt G2, Waters, Manchester, UK) MS system. The nLC column was eluted with a gradient of 0.1% formic acid in water (solution A) and acetonitrile (solution B). Samples were separated using the following gradient: 1%–12% B for 1 min; 12%–40% B for 16 min; 40%–85% B for 2 min; 85%–100% B for 2 min; and 1.0% B for 10 min. The autosampler was maintained at 10 °C. All analyses were performed in a data-dependent analysis (DDA).

### 4.6. Venom Gland RNA Extraction and Library Construction

Two Female *P. verdolaga* specimens (from Antioquia Province in the locality of La Estrella-Pueblo Viejo, Colombia) were anesthetized under CO_2_. Venom glands were excised and placed in plastic vials containing TRIzol^®^ reagent (ThermoFisher Scientific, Waltham, MA USA). Total RNA was extracted following the manufacturer’s protocol. RNAquality and concentration were measured using the bioanalyzer capillary system (Bioanalyzer Agilent 2100, Agilent, Santa Clara, CA, USA). mRNA was purified using the kit, and library preparation was carried out using the Illumina mRNA TruSeq kit v2. A 100 bp pair-end library was sequenced in a Hiseq 2500 instrument. Reads were cleaned using PRINSEQ-LITE (v0.2.) using default settings [44]. Genetic accession was carried out under contract 155 signed by the University of Antioquia with the Environmental Ministry of the Republic of Colombia.

### 4.7. De Novo Transcriptome Assembly and Gene Annotation

De novo assembly of transcripts was done using the TRINITY (v2.) assembler package under default settings [45]. Transcriptome assembly quality was assessed based on the calculated E90N50 contig length and Benchmarking Universal Single-Copy Orthologs (BUSCO) annotation [46]. Sequence reads were aligned back to the reference transcriptome using Bowtie2 [47], and RNA-seq by expectation maximization (RSEM) [48] was used to estimate transcript abundance for all transcripts as transcripts per million (TPM).

After assembly, all contigs/singlets were translated in six frames (16), and the Trinotate pipeline [45] was used to annotate the transcriptome. Briefly, homology searches were performed against the protein sequences contained in Genbank and UniProt databases using BLASTX with an *e*-value cutoff of 1 × 10^−5^. HMMER and Pfam databases were used to predict protein domains contained within each transcript. Presence of a signal peptide was determined using SignalP version 4.0. The resulting annotation information was then combined and pooled into an SQLite database. Also, to identify transcript homologs to spider toxins, BLASTX and tBLASTN programs (Sweet Version 2.28) were used. Cleaving signals for each transcript were predicted using the standalone tool and Tox|Note [22], which uses a combination of SignalP v4.1 [49] and an HMM to predict signal and propeptide sites, respectively. After the prediction of putative cleavage sites, mature peptides were aligned using the Clustal omega program [50]. Fasta36 (20) was used perform multiple high-scoring local alignments between the MS/MS sequences and the translated transcripts.

### 4.8. Nomenclature

Peptides and proteins were named following the nomenclature proposed by King et al. [51], with some modifications for proteins (masses above 20 kDa) i.e., protein group, followed by the isoform number and the species name. Names for all peptides and proteins annotated from the transcriptome were generated automatically using the Tox|Name module in the Tox|Note pipeline [22]. Spider taxonomic names were assigned according to the World Spider Catalog v20.0 (available at: https://wsc.nmbe.ch/) [21].

### 4.9. Protein Domain Searching and Sequence Logos

A total of 26 mature peptides were screened against online software (http://web.expasy.org/blast/) using the Blast program with an *e*-value cutoff set to <10^−5^ to identify similar sequences and putative functions of the new peptides. In order to identify the domain location within the query sequence, a multiple sequence alignment was performed among query and homology sequences, according to their cysteine pattern using the Geneious software package (v8.1.9) [52]. Resulting alignments were manually curated using GeneDoc [53]. Logo analysis of consensus sequences derived from alignments were generated using WEBLOGO (v2.8.2 https://weblogo.berkeley.edu/).

### 4.10. Disulfide Bond Prediction

Disulfide bonds were predicted using the Cysteines Disulfide Bonding State and Connectivity Predictor (DISULFIND http://disulfind.dsi.unifi.it/), a web-based tool for disulfide engineering in proteins [54].

### 4.11. Calcium Influx Assays

Five milligrams of *P. verdolaga* venom was fractionated by rp-HPLC (1 mg per run, venoms A–E) as described above, and fractions were evaluated for bioactivities using calcium influx imaging assays and the FLIPR^Tetra^ instrument (Molecular Devices, CA, USA) [15,55]. Briefly, hCa_V_2.2 and hNa_V_1.7 responses were evaluated in neuroblastoma SH-SY5Y maintained in Roswell Park Memorial Institute (RPMI) media supplemented with 15% FBS and 2 mM L-glutamine. The hCa_V_3.2 responses were evaluated using recombinant hCa_V_3.2 expressed in HEK293T cells maintained in Dulbecco’s modified Eagle’s medium (DMEM) supplemented with 10% FBS and 750 μg/mL geneticin. Cells were seeded at 40,000 and 10,000 per well for SH-SY5Y and HEK293T cells, respectively, and cultured in a humidified atmosphere for 48 h before the assay. Media was removed, and 20 μL of Calcium 4 dye (Molecular Devices) prepared in an assay buffer—(in mM) 140 NaCl, 11.5 glucose, 5.9 KCl, 1.4 MgCl_2_, 1.2 NaH_2_PO_4_, 5 NaHCO_3_, 1.8 CaCl_2_, and 10 HEPES (pH 7.4)—was added to the wells. Cells were incubated for 30 min at 37 °C 5% CO_2_. Ca^2+^ fluorescence responses were recorded at an excitation of 470–495 nm and emission of 515–575 nm for 10 s to set the baseline, at 600 s after addition of 20% of each venom fraction reconstituted in assay buffer, and for a further 300 s after addition of 90 mM KCl CaCl_2_ for Ca_V_2.2 activation, 40 mM KCl CaCl_2_ for hCa_V_3.2 activation, and 3 μM veratridine and 30 nM of the scorpion toxin OD1 for hNa_V_1.7 activation. For the hCa_V_2.2 assay, nifedipine at 10 μM was added to the Calcium 4 dye to inhibit endogenous hCa_V_1 responses. The controls for the inhibition of Ca^2+^ fluorescence responses were CVID at 1 μM for hCa_V_2.2, TTA-A2 at 50 μM for hCa_V_3.2, and tetrodotoxin (TTX) at 1 μM for hNa_V_1.7 responses. Data were normalized against the baseline (*F/F_0_*) and fluorescence responses plotted using GraphPad Prism 7. Venom fractions showing bioactivity were submitted to mass spectrometry analysis using matrix-assisted laser desorption/ionization time-of-flight mass spectrometry (MALDI-TOF MS) using a 4700 Proteomics Bioanalyser Model (Applied Biosystems, CA, USA). Peptides dissolved in water were mixed 1:1 (*v*/*v*) with matrix (7 mg/mL α-cyano-4-hydroxy-cinnamic acid in 50% ACN) and mass spectra acquired in positive reflector mode. Reported masses are for monoisotopic M + H^+^ ions.

### 4.12. Data Deposition

Metadata and annotated nucleotide sequences were deposited to the European Nucleotide Archive (ENA) under accessions: PRJEB21288/ERS1788422/ERX2067777-ERR2008012.

## 5. Conclusions

*P. verdolaga* venom is a rich source of cysteine-rich peptides that form cystine knots, which can affect vertebrate Ca_V_ and Na_V_ channels. Transcriptomic analysis showed the presence of at least 265 novel compounds. The venom content is similar to other Theraphosidae spiders like *G. rosea*, *C. schmidti* (*H. schmidti*), *C. guangxiensis,* and *C. hainanus* (*H. hainanum*).

## Figures and Tables

**Table 1:** Assignment of the rp-HPLC fractions from of *P. verdolaga* venom, with protein families matching the nESI-MS/MS collision-induced dissociation of peptide ions generated by in-gel digestion and the respective homologous venom gland transcript. Transcript abundance estimation is supported by transcripts per million (TPM) values. Cysteine residues are carbamidomethylated. Apparent molecular masses (Mapp, in kDa) were estimated by Tris-Tricine gel of β-mercaptoethanol-reduced (▼) samples.

rp-HPLC Fraction Number/Tris-Tricine Band	Mapp (kDa)	m/z	z	Peptide Sequence	Score	Best Transcriptomic Match	TPM
**21-22/a-b_1**	6–10▼	444.36	2	K.IKLCLKI.-	40%	c7142_g1_i1	27806.19
**21-22/a-b_2**		773.98	2	-.IFECVFSCDIEK.E	100%	c7142_g1_i1	27806.19
**21-22/a-b_3**		618.64	4	-.IFECVFSCDIEKEGKPCKPK.G	100%	c7142_g1_i1	27806.19
**21-22/c_1**	10▼	579.10	2	CVFSCDXEK	81.9%	c7142_g1_i1	27806.19

**Table 2:** *P. verdolaga* cysteine-rich proteins grouped by cysteine pattern.

Superfamily	Cysteine Pattern	Number of Cysteines	Mature Peptide Length
SF1	C-C-**CC**-C	5	54
SF2	C-C-C-**CC**-C	6	48
SF3	C-C-**CC**-C-C-C	7	43–84
SF4	C-C-**CC**-C-C-C-C	8	44–74
SF5	C-C-C-**CC**-C-C-C	8	61–65
SF6	C-C-C-C-**CC**-C-C	8	43–71
SF7	C-C-C-C-C-**CC**-C	8	76–80
SF8	C-C-**CC**-C-C-C-C-C-C	10	67–88

## Supplementary Materials

The following are available online at https://www.mdpi.com/2072-6651/11/9/496/s1. Supplementary File S1. Trinity assembly summary statistics of de novo reference transcriptome for venom gland of *Pamphobeteus verdolaga*. Base pair length distribution of transcripts from *Pamphobeteus verdolaga* venom gland. E90N50 statistic for de novo reference transcriptome of *Pamphobeteus verdolaga*. Supplementary File S2. Annotation of 16,030 transcripts of the venom gland of *Pamphobeteus verdolaga*.
