# Supplementary material for: Venomic, Transcriptomic, and Bioactivity Analyses of Pamphobeteus verdolaga Venom Reveal Complex Disulfide-Rich Peptides That Modulate Calcium Channels
Source: Toxins (Basel). 2019 Aug 27;11(9):496. doi: 10.3390/toxins11090496 (PMC6784019; doi:10.3390/toxins11090496)
Supplement: Supplementary file 1 [file toxins-11-00496-s001.zip › toxins-543942-SI/toxins-543942-supplementary - publish.docx]

Supplementary Materials: Venomic, Transcriptomic and Bioactivity Analysis of *Pamphobeteus verdolaga* Venom Reveal Complex Dissulfide Rich Peptides that Modulate Calcium Channels

Sebastian Estrada-Gomez, Fernanda Caldas Cardoso, Leidy Johana Vargas-Muñoz, Juan Carlos Quintana-Castillo, Claudia Marcela Arenas Gómez, Sandy Steffany Pineda and Monica Maria Saldarriaga-Cordoba

Profiling the peptide content of *Pamphobeteus verdolaga* venom

**Table S1.** Trinity assembly summary statistics of de novo reference transcriptome for venom gland of *Pamphobeteus verdolaga*.

| **Parameter** | **Number** |
| --- | --- |
| Total number of high quality assembled paired-end reads | 46,598,494 |
| Total trinity transcripts | 78088 |
| Total trinity 'genes' | 68,402 |
| Average 'genes' length (pb) | 559 |
| Total GC count (%) | 39.19 |
| N50 | 849 |
| Longest contig (bp) | 13,249 |
| Shortest contig | 201 |
| Number of contigs 200–999 bp | 67,124 |
| Number of contigs 1000–4999 bp | 10,717 |
| Number of contigs 5000–9999 bp | 242 |
| Number of contigs 10,000–13,299 bp | 5 |
| Number of predict ORF (transdecoder) | 16,042 |


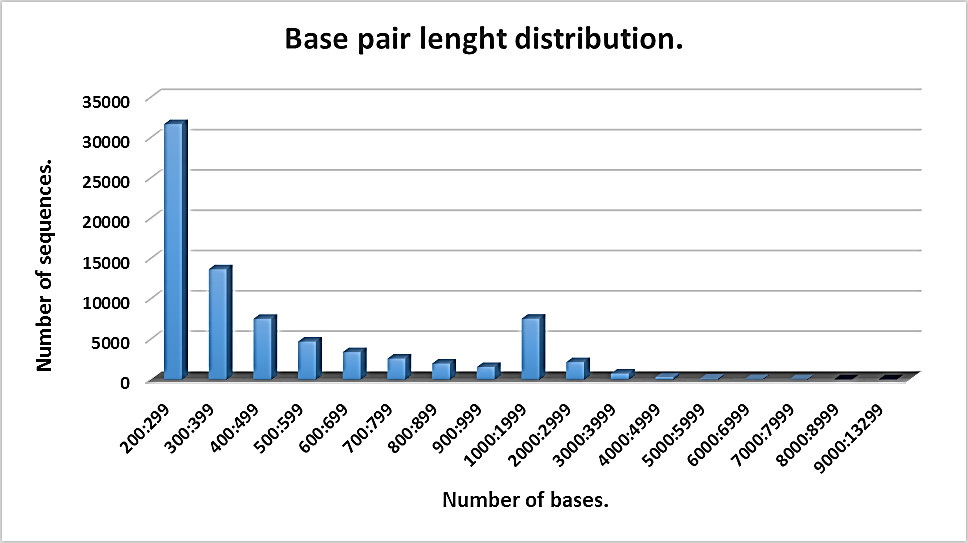


**Figure S1.** Base pair length distribution of transcripts from *Pamphobeteus verdolaga* venom gland.


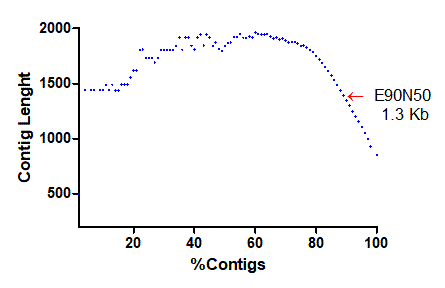


**Figure S2.** E90N50 statistic for de novo reference transcriptome of *Pamphobeteus verdolaga*. The reference transcriptome has an E90N50 of ̴1.3 kb (red arrow).
